# Participant-Independent Recognition of 22 Upper-Body Movements Using Wearable IMUs: A Controlled Pilot Study Toward Fine-Grained Industrial HAR

**DOI:** 10.3390/s26185835

**Published:** 2026-09-15

**Authors:** Chih-Feng Cheng, Chiuhsiang Joe Lin, Qin-Xuan Hu

**Affiliations:** 1 Department of Industrial Management, Asia Eastern University of Science and Technology, No. 58, Sec. 2, Sichuan Rd., Banqiao District, New Taipei City 220303, Taiwan; 2 Department of Industrial Management, National Taiwan University of Science and Technology, No. 43, Sec. 4, Keelung Rd., Da’an Dist., Taipei City 106335, Taiwan; cjoelin@mail.ntust.edu.tw (C.J.L.); rubyhu8888@gmail.com (Q.-X.H.)

**Keywords:** human activity recognition, inertial measurement units, time-series motion recognition, participant-level holdout evaluation, controlled movement recognition

## Abstract

**Highlights:**

**What are the main findings?**

- Using models developed from 13 participants, the best RF configuration achieved an aggregate accuracy of 0.950 and an F1 score of 0.939 when evaluated on three entirely unseen challenge-holdout participants whose observed execution patterns and fluency differed comparatively from those of the model-development participants.
- Despite this strong participant-independent aggregate performance, class-level reliability was not uniform; the most prominent errors remained concentrated in S18–S19, S4–S11, and S12–S14.

**What are the implications of the main findings?**

- The challenge-oriented participant-independent result supports the feasibility of transferring wearable-IMU motion recognition to unseen individuals with comparatively different execution characteristics, an important methodological prerequisite for future AI-assisted work study and human–robot collaboration.
- However, the localized class-specific errors show that strong aggregate performance does not guarantee uniformly reliable recognition of all operationally distinct movements; future industrial systems may therefore require class-resolved validation and, where necessary, additional contextual or temporal information.

**Abstract:**

Future industrial human activity recognition (HAR) may require discrimination among many operational elements, including movements with partially overlapping kinematics. This controlled pilot study characterized recognition across 22 upper-body movement classes with varying structural similarity and examined classifier, signal scaling, and temporal window length. Sixteen adults performed the movements with an XSENS motion-capture system; eight upper-limb inertial measurement units provided 80 synchronous time-series channels. Thirteen participants were used for model development, and three whose observed execution patterns differed comparatively from the remainder were deliberately reserved as a challenge-oriented holdout. Support vector classifier (SVC), random forest (RF), Gaussian naive Bayes (NB), and long short-term memory (LSTM) models were evaluated with min–max or maximum-absolute scaling and 62-, 93-, or 124-frame windows. RF with maximum-absolute scaling and a 124-frame window achieved the best aggregate holdout performance (accuracy = 0.950; F1 = 0.939). Importantly, this performance was obtained on three entirely unseen participants who were deliberately reserved because their observed execution patterns and fluency differed from those of the model-development participants, providing a controlled, challenge-oriented test of transfer across inter-individual execution variability. Nevertheless, strong aggregate performance did not translate into uniform class-level reliability, and prominent errors remained concentrated in specific movement pairs. These findings provide empirical evidence for both the participant-independent transfer capability and the class-specific limitations of motion-only recognition, supporting its role as a methodological precursor to future AI-assisted work study and human–robot collaboration rather than as evidence of end-to-end industrial HAR.

## 1. Introduction

Human activity recognition (HAR) in industrial contexts can be viewed as a multivariate time-series pattern-recognition problem in which sequential sensing data are used to infer the activity being performed. Unlike many activities of daily living, industrial work is commonly organized as a standard operating procedure (SOP) composed of consecutive operational elements or sub-actions. A longer-term objective of industrial HAR is therefore to identify operational elements from evolving motion features with sufficient granularity to support process understanding and subsequent decision making [1,2].

This capability connects contemporary artificial intelligence (AI) with industrial-engineering work study. Motion and time study decomposes work into meaningful elements and examines their sequence and duration; automatic recognition could eventually reduce part of this observation burden and support work-method analysis, training, process review, and ergonomics [1,2,3]. These applications remain prospective in this study because the experiment used controlled fundamental movements rather than complete industrial operations.

Human–robot collaboration (HRC) provides another prospective application context. Collaborative systems can benefit from reliable estimates of a worker’s current action or process state, but practical deployment must cope with subject variation, temporal transitions, sensing constraints, and operational context [4,5]. This study addresses only the controlled motion-recognition component of this broader problem.

Recent industrial HAR studies have addressed sequential motion recognition, assembly-action recognition and segmentation, process monitoring, and occupational assessment [5,6,7,8,9]. Many application studies nevertheless evaluate relatively small task-specific vocabularies, whereas practical procedures can contain numerous operational labels. As label vocabularies broaden, aggregate multiclass accuracy alone may not reveal whether individual movement classes remain reliably separable. Characterizing both global performance and class-resolved error structure is therefore important before end-to-end deployment is considered.

A related challenge is that inter-class similarity is heterogeneous. Some operational labels can be separated by distinct body segments or trajectories, whereas others differ only in laterality, dominant segment, orientation, amplitude, or workspace context. Prior industrial studies report ambiguity among similar actions [5,6,7], but class count and inter-class similarity are distinct dimensions of difficulty and should not be conflated.

This study therefore uses a controlled set of 22 fundamental upper-body movement classes organized into four descriptive task groups. The set includes both readily distinguishable movements and structurally similar cases, allowing aggregate recognition performance to be interpreted together with class-resolved error patterns. This design permits a controlled examination of whether strong global recognition performance is accompanied by uniform class-level reliability or instead coexists with localized confusion. The experiment is a methodological precursor to industrial HAR rather than a reproduction of a complete manufacturing process, and its participant-level holdout design evaluates transfer to individuals excluded from model development without claiming population-representative validation.

Accordingly, this study has two objectives. First, it characterizes participant-level holdout classification performance and the class-resolved error structure across the controlled 22-class movement set. Second, it examines how the evaluated classifier, scaling strategy, and segmentation-window setting affect recognition performance. The contribution is empirical rather than algorithmic. Specifically, the participant-independent challenge-holdout design provides a controlled test of whether a wearable-IMU recognition pipeline developed from one group of participants can retain substantial discriminative capability when applied to entirely unseen individuals exhibiting comparatively different execution patterns and fluency. The study further examines whether strong aggregate transfer performance is accompanied by uniformly reliable class-level discrimination. Together, these two perspectives provide evidence about both the transfer capability and the localized limitations of motion-only recognition before extension to AI-assisted work study, human–robot collaboration, and richer industrial procedures.

## 2. Related Works

The literature relevant to this pilot study is summarized from four complementary perspectives: HRC and assembly assistance; AI-assisted work study and process monitoring; transferable temporal/spatial recognition methods; and the resulting research gap. The emphasis is on methodological issues that bear directly on the controlled experiment rather than on broad industrial deployment claims. Table 1 provides a concise comparison of representative studies.

### 2.1. HRC and Assembly-Process Assistance

For HRC, activity recognition can serve as a perception layer linking observed human motion to the current state of collaborative work. Strong unseen-subject performance has been reported for small vocabularies of manufacturing primitives [4]. When recognition is extended to untrimmed assembly sequences, however, variable duration, ambiguous transitions, and neighboring actions with similar motion patterns increase the difficulty [5,7].

Spatial representation and contextual information become particularly important when different labels involve the same limbs or similar temporal profiles. Direction-sensitive skeletal representations can help separate subtle assembly motions [8], while cross-modal fusion of skeletal and red-green-blue (RGB) information can improve discrimination of highly similar assembly actions [6]. These findings motivate cautious interpretation of motion-only results when class similarity is high.

Taken together, HRC and assembly studies suggest that recognition difficulty depends not only on the number of labels but also on temporal variability, spatial overlap, and the availability of contextual cues. The present experiment isolates the motion-information layer under controlled segmentation, rather than attempting full collaborative-process monitoring.

### 2.2. AI-Assisted Work Study, SOP Compliance, Safety, and Occupational Assessment

From an industrial-engineering perspective, sequential HAR could eventually support work study by assigning operational labels to observed motion and helping analysts review sequence, duration, and deviations from a learned work method. Participant-independent studies in logistics and manual assembly provide direct methodological precedents for this objective [1,2], while continuous process monitoring additionally requires reliable action-boundary and process-state inference [7].

Temporal structure is particularly relevant when execution speed and duration vary. Dynamic time warping combined with *k*-nearest neighbors (DTW + KNN) can compare evolving sequences directly and has been used for SOP-defined assembly sub-actions [2]. At the same time, accurate classification of pre-segmented movements does not constitute online process monitoring; automatic segmentation and handling of pauses or redundant behaviors remain separate requirements.

### 2.3. Transferable Methods for Sequential and Fine-Grained Motion Recognition

A first transferable requirement is the preservation of temporal structure. Fixed windows may contain incomplete or phase-shifted portions of the same action, whereas sequence-alignment approaches can accommodate timing variation [2,12]. This motivates evaluating multiple temporal windows and considering adaptive or alignment-based methods in future work.

A second requirement is the representation of spatial relationships among body segments. Large multiclass skeleton models demonstrate that high class counts are computationally feasible [10], but industrially adjacent labels may differ only modestly in direction, joint configuration, or amplitude. Direction-sensitive representations are therefore especially relevant for subtle movements [8].

A third requirement is context. When the operational label is not uniquely determined by body motion, object identity, tool state, location, or preceding process state can provide information that motion alone lacks. Sequential object features in fine-grained recognition [11] and multimodal assembly recognition [6] illustrate this principle. The present experiment deliberately does not add such contextual cues, so its results delimit only the motion-recognition layer.

### 2.4. Synthesis of the Research Gap and Positioning of This Study

Across the reviewed literature, industrial HAR has progressed from small sets of manufacturing primitives toward sequential sub-action recognition, temporal segmentation, multimodal assembly monitoring, and work assessment. Two methodological questions remain relevant to this pilot study: how a practical wearable-motion workflow behaves when the number of labels increases, and whether aggregate multiclass performance adequately represents class-level separability when errors may concentrate in particular movement pairs.

Most industrial application studies summarized in Table 1 use task-specific label sets smaller than the 22 classes evaluated here, while similar-action ambiguity is generally encountered within a particular process rather than quantified as a universal property of the complete class set. Accordingly, this study does not assume that all 22 classes are equally similar and does not define a post hoc “high-similarity” subset.

Instead, the study characterizes aggregate performance across 22 classes, together with class-resolved error patterns, using a participant-level challenge holdout. The scientific value of this design lies in two complementary aspects. First, the challenge-oriented participant-level holdout evaluates transfer to entirely unseen individuals whose observed execution characteristics differed comparatively from those represented during model development. Second, aggregate performance across the 22 classes is interpreted alongside class-resolved error patterns, allowing for joint evaluation of global recognition capability and localized recognition limitations rather than treating aggregate accuracy as evidence of uniform class-level reliability.

## 3. Materials and Methods

### 3.1. Participants and Ethical Considerations

Sixteen adults participated in the controlled fundamental-movement experiment. The sample comprised eight men (mean height = 174.6 cm; standard deviation [SD] = 5.3 cm) and eight women (mean height = 161.6 cm; SD = 4.7 cm), with ages ranging from 18 to 50 years. All participants were reported to have no impairments affecting movement of the upper or lower limbs. Sensor placement and system calibration required approximately 15 min, followed by approximately 15 min of experimental movement trials. The modest sample size is interpreted in the context of an exploratory pilot study rather than population-level validation.

### 3.2. Apparatus and Software

Upper-body kinematic data were acquired using a wearable XSENS (Xsens Technologies B.V., Enschede, The Netherlands) motion-capture system. Thirteen IMUs were attached to the participant’s upper body and communicated wirelessly with a base station connected to a 15.6-inch ASUS (ASUSTeK Computer Inc., Taipei, Taiwan) notebook computer. Signals were sampled at 60 Hz. The acquisition system recorded orientation as a four-element quaternion, three-axis acceleration, and three-axis angular velocity. Data were initially organized in MVN Analyze (v2025.0) and subsequently exported to Microsoft Excel 2019 for data management. Machine-learning analyses were performed on a 64-bit Windows 10 desktop computer equipped with an Intel Core i7-9700 3.0 GHz processor and 16 GB of memory.

**Figure 1:**
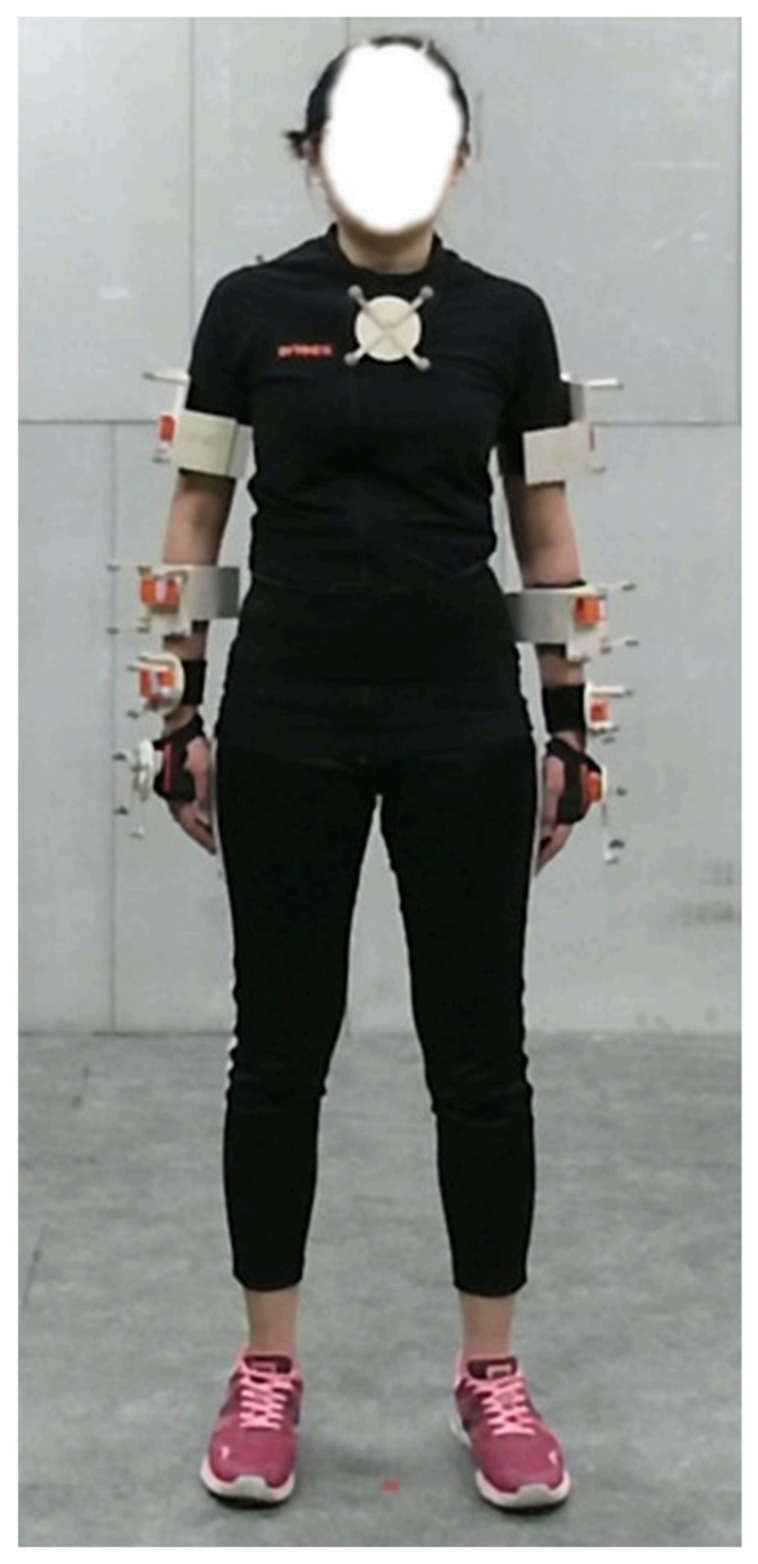
Upper-body XSENS sensor placement and the neutral reference posture with both arms relaxed alongside the body.

Although 13 IMUs were worn to support system calibration and full upper-body tracking, the classification analysis used the eight IMUs located bilaterally at the hands, wrists, elbows, and upper arms because the target movement set was dominated by upper-limb activity. Placement was adjusted to participant morphology while favoring locations with less pronounced muscle-contour change to reduce motion artifacts. Figure 1 shows the sensor placement and neutral reference posture. The study did not evaluate whether fewer sensors would preserve classification performance.

### 3.3. Experimental Tasks and Procedure

The experiment was designed to capture a broad range of fundamental upper-body movement patterns. Twenty-two movement classes (S1–S22) were defined to span upper-arm, forearm, head, trunk, and shoulder motions. Participants were not required to complete each movement within a fixed duration; instead, they moved at a natural pace, resulting in variable-length recordings. Individual movement executions typically lasted approximately 1–3 s. Before and after each movement, participants adopted a neutral standing posture with both arms relaxed alongside the body for 1–2 s. This common reference posture was intended to reduce the preprocessing variability associated with inconsistent starting and ending configurations.

For experimental organization, the 22 movement classes were divided into four groups according to the principal body segment involved: Task 1 comprised S1–S7 (upper-arm movements), Task 2 comprised S8–S14 (forearm movements), Task 3 comprised S15–S19 (head and trunk movements), and Task 4 comprised S20–S22 (shoulder movements). These groups were descriptive/organizational and were not analyzed as an independent factor; the classifier retained all 22 S-codes as separate target classes. The set contained structurally similar cases, including corresponding upper-arm/forearm movement directions and left/right trunk rotations, but no quantitative similarity threshold was defined.

**Figure 2:**
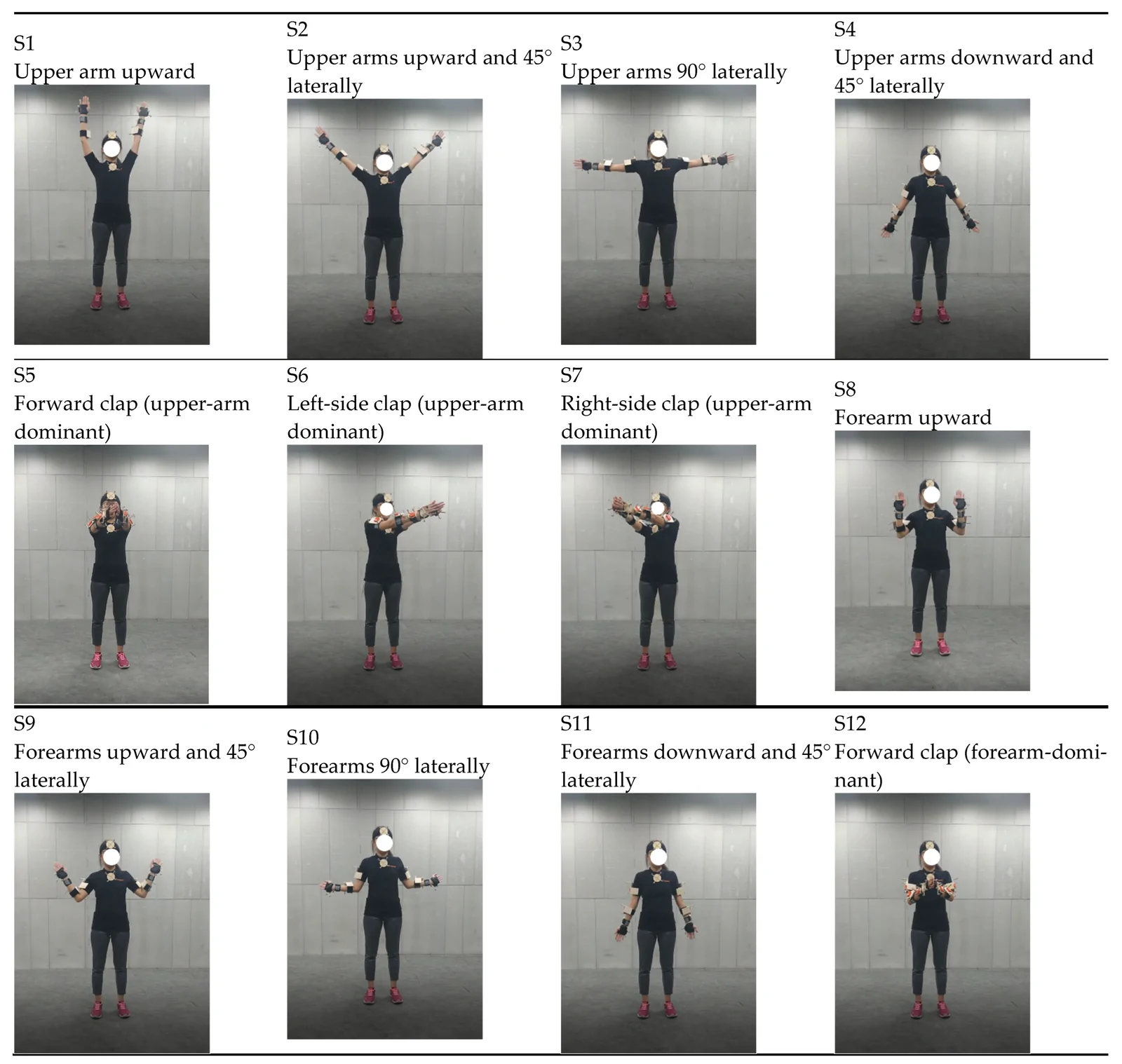

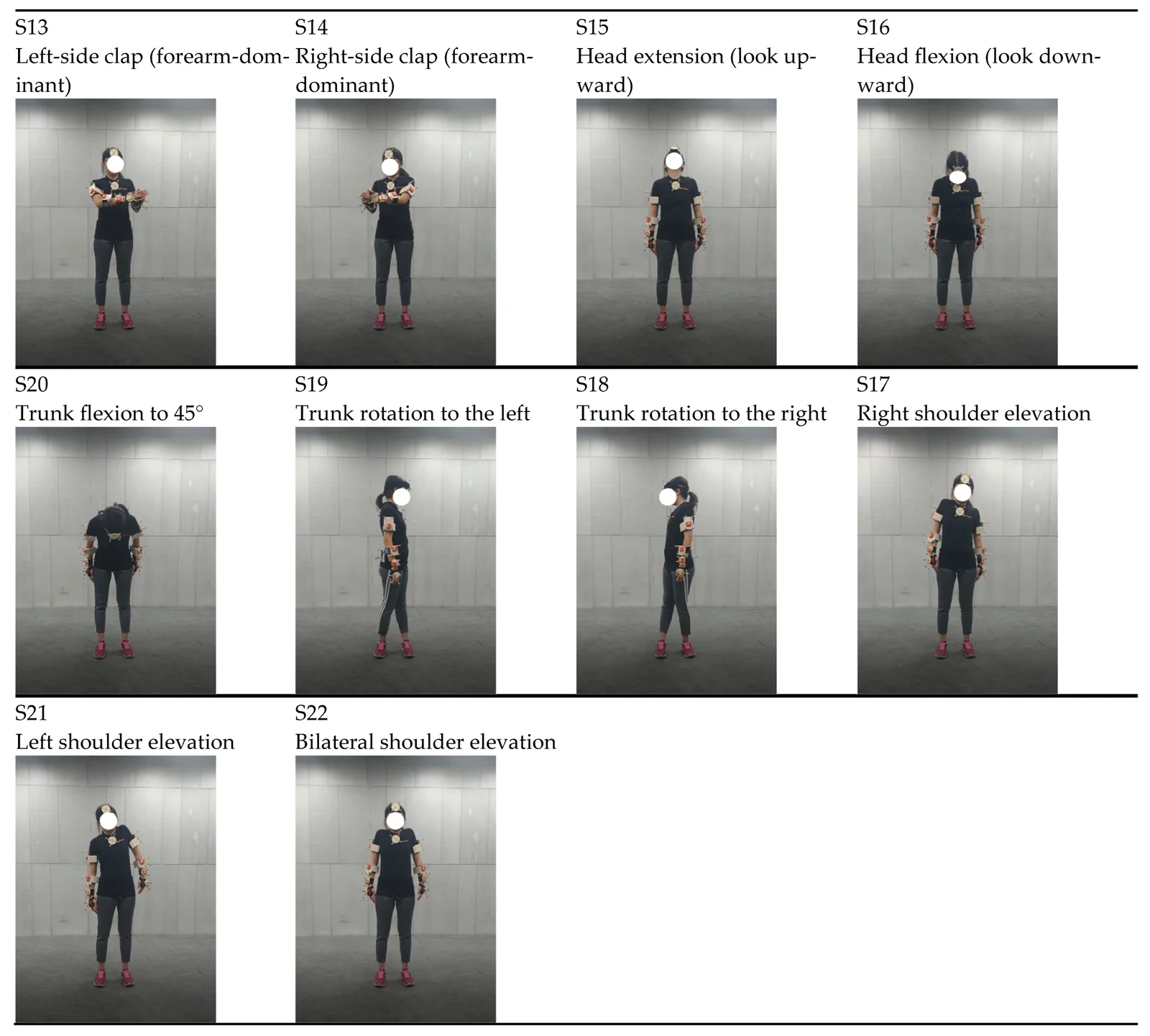
Representative postures for the 22 fundamental movements (S1–S22), with labels indicating the intended movement direction and dominant body segment where applicable.

The 22 movement classes, defining body segments, and representative postures are summarized in Figure 2.

### 3.4. Data Acquisition and Preprocessing

For each of the eight upper-limb IMUs retained for analysis, 10 signal components were available: four quaternion orientation components, three acceleration axes, and three angular-velocity axes. Each movement sample was therefore represented by 80 synchronous continuous time-series channels (10 components × 8 IMUs).

Occasional sensor interruptions during data collection produced unavailable records, which were removed before segmentation and model development. The archived model-development dataset contains 279 movement recordings from 13 participants spanning the 22 classes, with a mean sample length of 185 frames (SD = 48.2), a minimum of 36 frames, and a maximum of 330 frames. Table 2 summarizes these verified model-development data. The archived records available for this revision do not contain a verifiable class-by-class count table for the segmented windows of the three holdout participants; therefore, no test-window counts were inferred retrospectively.

Two scaling strategies were compared because the signal channels had different numerical ranges. Min–max scaling mapped each variable to [0, 1], whereas maximum-absolute scaling divided by the maximum absolute value and preserved signal sign, yielding values within [−1, 1]. Previous gesture/activity-recognition studies have examined these scaling strategies [13,14], motivating their comparison here; this study does not assume that either strategy is universally beneficial. The transformations were implemented as follows:

*x*′ = (*x* − *x_min*)/(*x_max* − *x_min*) (1)

*x*′ = *x*/*max*(|*x*|) (2)

Time-series segmentation and feature extraction were performed using the Python package seglearn (v1.2.5) [15]. Consecutive windows overlapped by 75%. The mean model-development movement length was 185 frames, and three candidate windows were selected to represent approximately one-third, one-half, and two-thirds of this duration: 62 frames (approximately 1.03 s at 60 Hz), 93 frames (approximately 1.55 s), and 124 frames (approximately 2.07 s). When a movement execution was shorter than the target window, zeros were appended using numpy.pad until the required length was reached so that the execution was not discarded and equal-length LSTM inputs could be formed [16]. The minimum recorded length was 36 frames; consequently, the padded proportion could be substantial for an extremely short execution under the 124-frame setting. The influence of padding proportion was not evaluated independently. Table 3 summarizes the three window settings.

For the conventional machine-learning classifiers, each segmented window was transformed into 11 summary features using seglearn.transform.FeatureRep [15]. These descriptors are listed in Table 4. The LSTM model did not use this hand-crafted feature representation; segmented sequential data were supplied directly to the network so that representation learning and classification were performed jointly.

### 3.5. Classification Models and Training

Four supervised classifiers were compared: a linear support vector classifier (SVC); a random forest (RF); a Gaussian naive Bayes (NB); and an LSTM network. The intended participant-level allocation approximated an 80/20 model-development/holdout split: 13 of the 16 participants (12.8 rounded to 13) were used for model development and three were reserved for holdout evaluation. The holdout participants, denoted P1–P3, were deliberately selected during data collection because their observed movement execution and fluency appeared comparatively different from those of the remaining participants, creating a challenge-oriented rather than randomized holdout. No participant contributed windows to both sets. Accordingly, this design was intended as a controlled challenge-oriented assessment of participant-independent transfer across inter-individual differences in movement execution, rather than simply a random estimate of average unseen-subject performance. Because the holdout was deliberately selected, its results are interpreted as an exploratory stress test and not as an unbiased estimate of population-level generalization. The movement labels themselves did not distinguish standard from non-standard execution, so the experiment should not be interpreted as anomaly or SOP-conformance detection.

For RF, GridSearchCV from sklearn.model_selection was used during model development to select the number of trees (n_estimators) and maximum tree depth (max_depth). For SVC, linear, radial basis function (RBF), and sigmoid kernels were examined, with the linear kernel providing the best observed result. GaussianNB was used for NB. The LSTM was implemented in TensorFlow/Keras with 64 LSTM units, dropout = 0.1, three dense hidden layers of 256 units, the Adam optimizer (learning rate = 0.001), a batch size of 100, up to 300 epochs, and cross-entropy loss. Table 5 summarizes the classifier implementations and principal settings. The original analysis retained training-loss trajectories but not corresponding validation-loss histories in a form that could be reliably reconstructed; the training-loss figures were therefore removed from the revision rather than used as evidence of generalization.

### 3.6. Model Performance Evaluation

Classification performance was summarized using aggregate accuracy and the F1 score, with confusion matrices used for class-resolved diagnostics. Accuracy measures the proportion of all holdout predictions that are correct across the 22 classes, whereas F1 summarizes the precision–recall trade-off. The confusion matrices identify specific class pairs responsible for errors that cannot be diagnosed from scalar scores alone. No a priori performance threshold was specified, and no population-level significance test was conducted from the three deliberately selected holdout participants. For a given class, true positives (TP), false positives (FP), and false negatives (FN) were used in the metric definitions below.

*Accuracy* = *Number of correct predictions*/*Total number of predictions* (3)

*Precision* = *TP*/(*TP* + *FP*) (4)

*Recall* = *TP*/(*TP* + *FN*) (5)

*F*1 = 2(*Precision* × *Recall*)/(*Precision* + *Recall*) (6)

The same multiclass F1 aggregation setting was applied consistently to the reported models and participants; however, the archived record available for this revision does not specify the averaging convention. F1 is therefore reported descriptively as originally computed, alongside accuracy and class-resolved confusion patterns.

## 4. Results

### 4.1. Overall Classification Performance

The controlled experiment comprised 22 movement classes evaluated with four classifiers, three candidate window lengths, and two scaling methods. Table 6 summarizes the best reported holdout configuration for each classifier. Among the evaluated models, RF achieved the highest aggregate performance, with accuracy = 0.950 and F1 = 0.939 using maximum-absolute scaling and a 124-frame window. The corresponding model-training time was approximately 10.9 s. The best reported SVC, NB, and LSTM configurations achieved accuracies of 0.778, 0.663, and 0.644, respectively. Because the holdout participants were deliberately selected as a challenge set, these aggregate values characterize this pilot evaluation rather than population-average performance.

Training-time differences were substantial under the respective best configurations: SVC and NB required approximately 3–4 s, RF approximately 11 s, and the evaluated LSTM approximately 676 s. These values describe model-development cost only; inference latency was not measured and real-time suitability cannot be inferred from training time.

### 4.2. Effect of Scaling and Participant-Level Performance

Table 7 and Table 8 provide participant-level results for the best window reported for each classifier under min–max and maximum-absolute scaling, respectively. For RF and NB, for which the best window was unchanged across scaling methods, maximum-absolute scaling was associated with higher mean accuracy (RF: 0.833 to 0.950; NB: 0.514 to 0.663). SVC accuracy remained 0.778 under both scaling methods, with essentially unchanged F1 scores (0.710 and 0.709). LSTM also showed higher mean accuracy under maximum-absolute scaling (0.604 to 0.644); however, its best reported window changed from 93 to 124 frames, so this difference should not be attributed to scaling alone.

Under maximum-absolute scaling, RF showed the smallest descriptive between-participant variation across the three challenge-holdout participants, with mean accuracy = 0.950 (SD = 0.030) and mean F1 = 0.939 (SD = 0.010). NB and LSTM showed larger descriptive accuracy SDs of 0.194 and 0.145, respectively. Given the deliberate P1–P3 selection and small holdout size, these SDs are not interpreted as population-level estimates.

### 4.3. Confusion Patterns and Movement-Specific Errors

**Figure 3:**
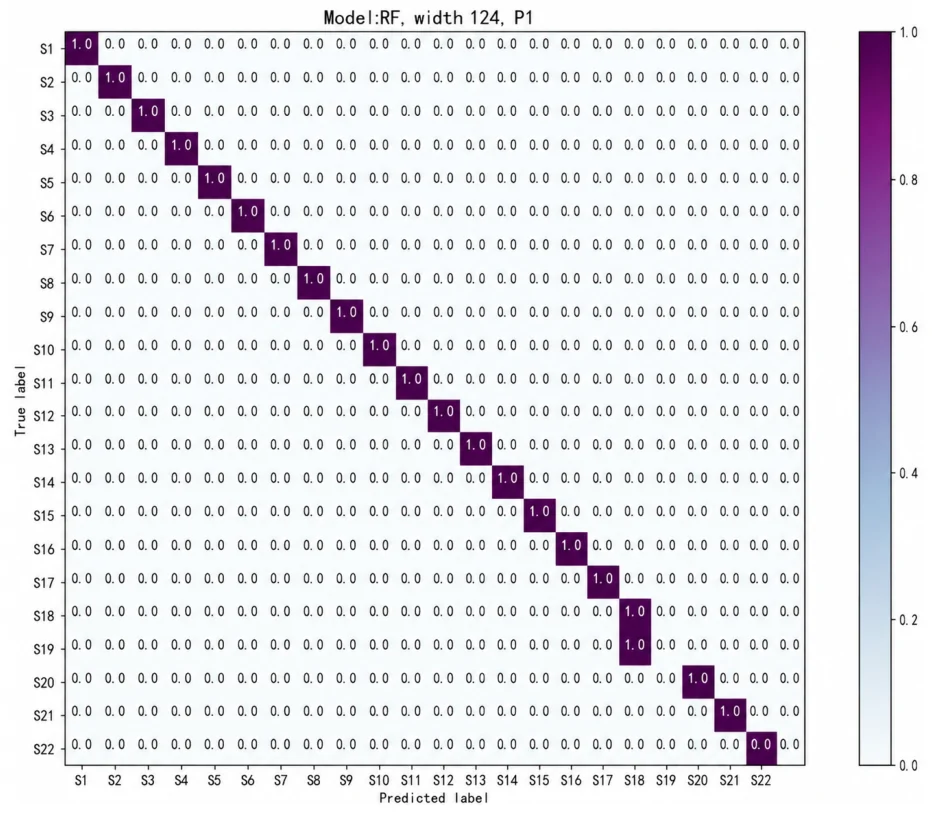
Confusion matrix for challenge-holdout participant P1 using RF with maximum-absolute scaling and a 124-frame window.

**Figure 4:**
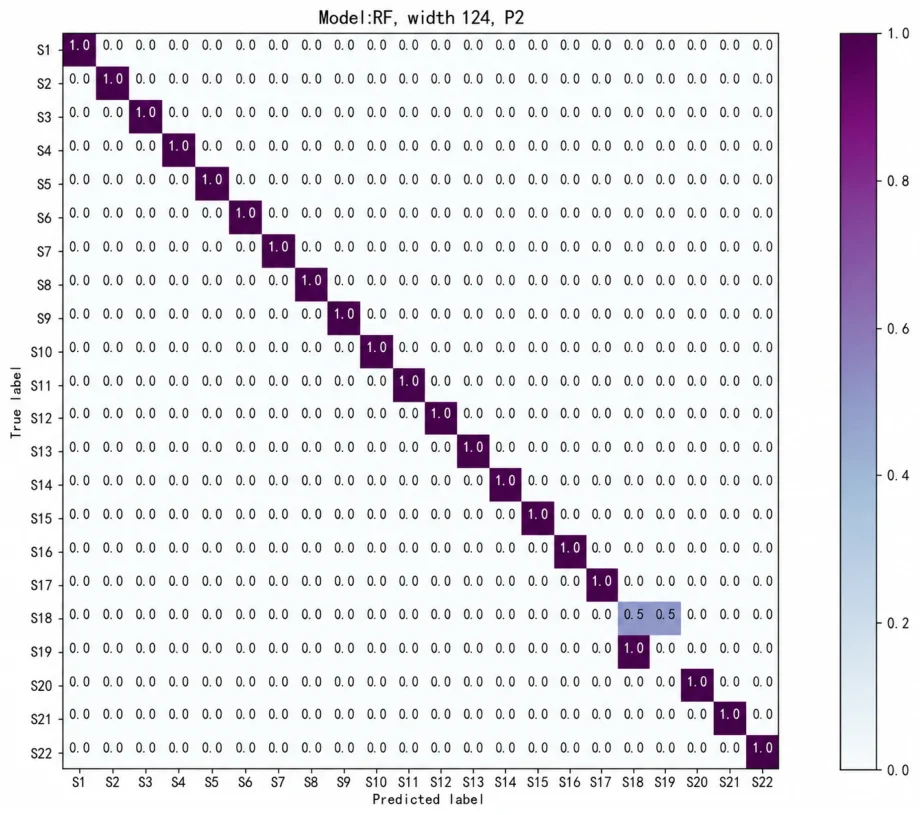
Confusion matrix for challenge-holdout participant P2 using RF with maximum-absolute scaling and a 124-frame window.

**Figure 5:**
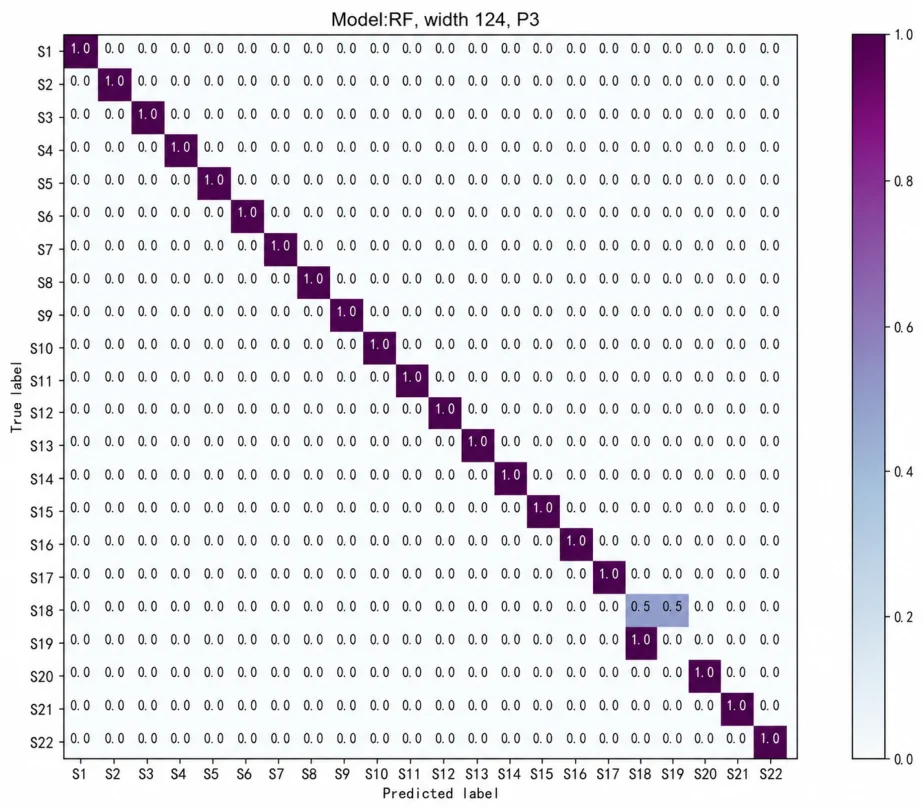
Confusion matrix for challenge-holdout participant P3 using RF with maximum-absolute scaling and a 124-frame window.

The confusion matrices for the three challenge-holdout participants (P1–P3) under the best overall RF configuration (maximum-absolute scaling, 124-frame window) are presented in Figure 3, Figure 4 and Figure 5. For P1 and P2, the most prominent reciprocal confusion occurred between left trunk rotation (S18) and right trunk rotation (S19). For P3, notable confusion occurred between the upper-arm-dominant downward 45° movement (S4) and the forearm-dominant downward 45° movement (S11), and between forward clap (forearm-dominant; S12) and right-side clap (forearm-dominant; S14). Thus, the aggregate 0.950 accuracy did not represent uniform performance across all class pairs.

**Figure 6:**
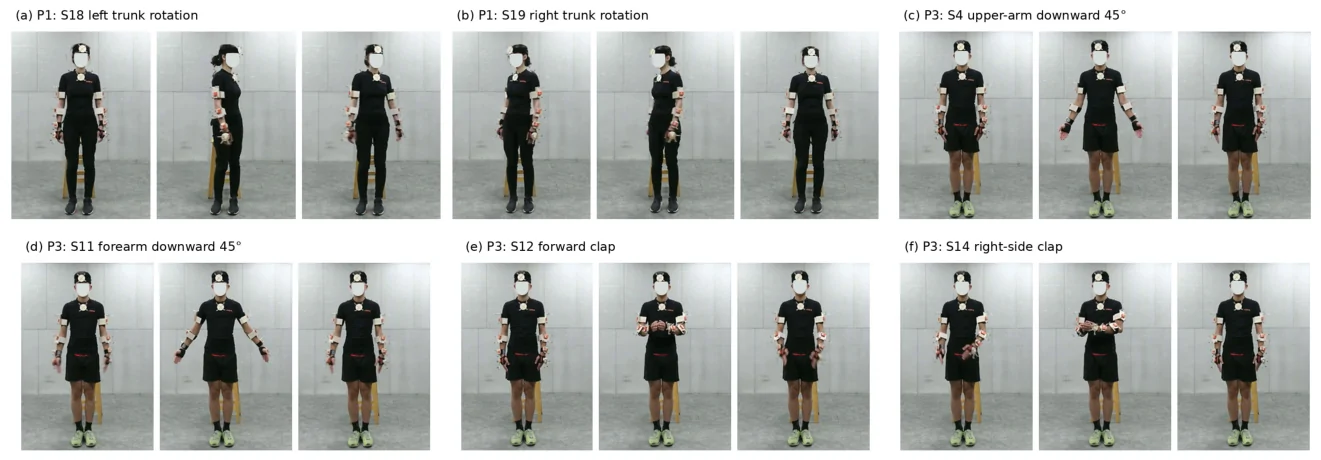
Qualitative three-stage movement sequences for prominent confusion pairs: (**a**) P1 S18 left trunk rotation; (**b**) P1 S19 right trunk rotation; (**c**) P3 S4 upper-arm-dominant downward 45° movement; (**d**) P3 S11 forearm-dominant downward 45° movement; (**e**) P3 S12 forward clap; and (**f**) P3 S14 right-side clap. These images illustrate recorded executions and are not a quantitative measure of kinematic similarity.

Qualitative inspection of the recorded movement sequences provided plausible, but not quantitatively verified, explanations for these errors. For P1, visible arm swing during the return from S18 or S19 to the neutral posture may have added motion patterns that reduced separability between the two trunk-rotation classes (Figure 6a,b). P2 also showed S18–S19 confusion without the same obvious arm swing. An ancillary archived RF analysis using a shorter 93-frame window yielded 98.6% accuracy for P2, with approximately 20% of S18 windows misclassified as S19 and the remaining movements classified correctly, suggesting that a single global window may not be optimal for every participant.

For P3, S4 and S11 both involved downward 45° arm configurations but differed in the intended dominant segment; the recorded S4 amplitude was visually limited, which may have reduced the distinction (Figure 6c,d). The S12–S14 confusion likewise involved forearm-dominant movements with relatively small displacement (Figure 6e,f). These examples show that prominent errors occurred among several qualitatively similar movement pairs. No pairwise kinematic-distance matrix or confusion-versus-similarity analysis was performed, so the observations should not be interpreted as quantitative evidence that similarity caused the errors.

## 5. Discussion

### 5.1. Principal Findings and Relation to the Research Objectives

The first objective yielded two complementary findings concerning participant-independent recognition. First, the best RF configuration, developed using data from 13 participants, retained strong aggregate discrimination when applied to three entirely unseen challenge-holdout participants whose observed execution patterns and fluency differed comparatively from those of the model-development group (accuracy = 0.950; F1 = 0.939). This provides controlled evidence that the learned motion representation can transfer across inter-individual execution variability. Second, this strong aggregate transfer did not imply uniform class-level reliability: the most prominent errors remained concentrated in S18–S19, S4–S11, and S12–S14. Together, these findings distinguish participant-independent transfer capability from class-specific separability and show why both matter when evaluating wearable-IMU HAR for finer-grained applications. Because P1–P3 were deliberately selected and only three participants formed the holdout, this finding should be interpreted as challenge-oriented pilot evidence rather than as population-representative generalization.

The second objective was to examine the effects of the evaluated classifier, scaling strategy, and temporal window. RF produced the best aggregate holdout performance among the four evaluated model families, while maximum-absolute scaling was associated with higher RF and NB performance under the same best window. The evaluated LSTM required substantially more training time and achieved lower accuracy in this dataset. These observations apply only to the present configurations; they do not establish universal superiority of RF, maximum-absolute scaling, or feature-based models over stronger temporal architectures.

Taken together, the experiment extends earlier participant-independent sequential-motion work [1,2] to a comparatively large controlled 22-class movement vocabulary. Its contribution is empirical rather than algorithmic: the results show that a strong participant-independent aggregate score can coexist with persistent class-specific failures within the same multiclass vocabulary. This distinction provides evidence of the capabilities and limitations of motion-only recognition that global metrics alone would obscure.

Because the classes were pre-defined fundamental movements rather than continuous industrial operations, the results are best interpreted as evidence of a methodological prerequisite for future industrial HAR, not as proof of end-to-end SOP monitoring or work-method conformity.

### 5.2. Effects of Preprocessing, Temporal Windowing, and Model Choice

Scaling showed the clearest descriptive influence for RF and NB. With the same best window, RF accuracy increased from 0.833 under min–max scaling to 0.950 under maximum-absolute scaling, and NB increased from 0.514 to 0.663, whereas SVC remained at 0.778. Because scaling preceded feature extraction, it also changed derived statistics such as mean, extrema, variance, and absolute energy. Preserving signed values under maximum-absolute scaling may have retained useful directional contrasts, but this mechanism was not tested directly and should be regarded as a dataset-specific hypothesis rather than a general rule.

Window length interacted with movement duration. The 124-frame window (approximately 2.07 s at 60 Hz) was the best reported setting for SVC, RF, and LSTM and may have captured a more complete movement signature than shorter windows. However, longer windows could also include return-to-neutral or compensatory motion, and P2 achieved 98.6% RF accuracy in an ancillary 93-frame analysis. In addition, executions shorter than the target window were zero-padded, and the effect of padding proportion was not tested. Adaptive, multi-scale, duration-aware, or alignment-based temporal processing is therefore a relevant next step.

The model ranking should likewise be interpreted in relation to the available data and implementation. RF can accommodate nonlinear interactions among high-dimensional window-level features, whereas Gaussian NB and a linear SVC impose different representational assumptions. The evaluated LSTM learned directly from sequences but was developed from 279 movement recordings across 13 participants; overlapping windows increased training instances without adding equivalent participant diversity. Its lower accuracy and longer training time therefore characterize this particular architecture and dataset, not deep temporal models in general. Training time also cannot be used as a proxy for inference latency.

### 5.3. Similar-Action Confusion and the Industrial Meaning of the Error Structure

The class-resolved results show that errors were structured rather than uniformly distributed. P1 and P2 confused left and right trunk rotation (S18–S19), while P3 showed errors between upper-arm- and forearm-dominant downward 45° movements (S4–S11) and between two forearm-dominant clapping movements (S12–S14). This concentration matters because applications requiring finer operational labels depend on reliable class-level discrimination, not just high aggregate correctness. These pairs are qualitatively similar in laterality, dominant segment, direction, or amplitude. However, the study did not compute a pairwise kinematic-distance matrix or test a confusion-rate-versus-similarity relationship; consequently, no causal or quantitative relationship between similarity and error rate is claimed.

This interpretation is consistent with prior industrial HAR findings that identify similar gestures, temporal ambiguity, and contextual insufficiency as practical difficulties [5,6,7,8]. This pilot contributes a complementary controlled observation: high aggregate discrimination was possible across 22 classes, while the remaining prominent errors clustered in several qualitatively similar pairs. Thus, aggregate multiclass accuracy and local class separability are not interchangeable descriptions of recognition performance. This does not mean that all 22 classes were equally difficult or uniformly “fine-grained.”

Participant-specific execution also appears relevant. Return-to-neutral arm swing, reduced movement amplitude, and timing variation can plausibly make nominally distinct classes more alike, although these factors were not experimentally isolated. For future industrial applications, motion may therefore need to be combined with object identity, tool state, workstation location, or SOP process state when different operational labels share strongly overlapping kinematics. Worker-specific calibration or adaptive temporal modeling may also improve robustness, but these possibilities require direct testing.

### 5.4. Comparison with Related HAR Studies and Methodological Positioning

Prior studies provide complementary rather than directly comparable evidence. Participant-independent recognition has been demonstrated for smaller logistics and manufacturing vocabularies [1,4], sequence alignment has improved recognition of SOP-defined sub-actions [2], and recent assembly studies address untrimmed segmentation, multimodal context, and directional spatial representation [5,6,7,8]. Because these studies differ in sensing modality, label definition, segmentation, and validation design, their reported accuracies should not be ranked directly against the present 0.950 aggregate holdout result.

This experiment isolates a narrower question: whether an established wearable time-series pipeline can separate a comparatively large, controlled movement vocabulary and whether its aggregate performance adequately represents class-level reliability. The results of this study show that these two views of performance can diverge: strong global accuracy can coexist with persistent pair-specific errors. The literature indicates how future work can extend this baseline through automatic segmentation, temporal alignment [2,10], stronger spatial models [8,11], contextual object/process information [6,12], and occupational/workflow evaluation [9]. Validation in realistic procedures remains necessary before industrial deployment claims are warranted.

### 5.5. Implications for AI-Assisted Work Study and Human–Robot Collaboration

From an industrial-engineering perspective, the results of this study support continued investigation of HAR as a future information-acquisition layer for work study rather than demonstrating such a system directly. A participant-independent classifier that can distinguish sufficiently fine operational elements could eventually support SOP review, worker training, process-deviation analysis, or ergonomic assessment. This experiment does not calculate standard times, reconstruct complete work cycles, or detect process conformance; it establishes only controlled motion-classification feasibility.

The challenge-oriented participant-independent design is particularly relevant to these prospective applications. In practical work-study or collaborative-manufacturing settings, a recognition model cannot generally assume that every worker will reproduce a reference movement with identical timing, amplitude, or fluency. In this experiment, we obtained model-development data from 13 participants, whereas P1–P3 were entirely unseen and deliberately reserved because their observed execution characteristics differed from those of the remaining participants. The strong aggregate RF performance in this holdout therefore provides controlled pilot evidence that wearable-IMU recognition can retain substantial discriminative capability across worker-to-worker execution variability. Such transfer is an important prerequisite if HAR is eventually to support work-method observation, training, or worker-state estimation in HRC. This should not be interpreted as automatic detection of incorrect work methods or SOP deviations, because the present labels did not distinguish standard from non-standard execution.

The same caution applies to HRC. A collaborative system requires reliable perception of worker state, but this experiment did not evaluate continuous inference, action-boundary detection, safety-related failures, or end-to-end latency. RF required less training time than the evaluated LSTM, but training time is distinct from inference latency and cannot establish real-time suitability. Deployment studies should report sensing, preprocessing, inference, and decision latency together with task-level safety and reliability outcomes.

Wearable inertial sensing can complement camera-based monitoring because it is less affected by visual occlusion and does not record identifiable scene imagery, but multiple IMUs impose setup and calibration burdens. In this study, 13 IMUs were worn and eight upper-limb IMUs were analyzed; sensor-ablation performance was not evaluated. Placement may also vary with body morphology despite efforts to avoid locations with pronounced muscle-contour change. Future work should therefore examine placement robustness and reduced-sensor configurations before routine industrial use.

### 5.6. Limitations and Future Research

First, ecological and temporal validity are limited. The 22 labels were controlled fundamental movements bracketed by a neutral posture rather than continuous industrial operations involving tools, products, workspace constraints, uncertain boundaries, pauses, and natural transitions. Future studies should embed the recognition framework in real procedures and evaluate automatic segmentation, untrimmed streams, and the contribution of object, tool, location, and process-state context.

Second, validation was exploratory. Sixteen adults participated; 13 were used for model development and three deliberately selected participants with comparatively different observed execution patterns and fluency formed a challenge-oriented holdout. This non-random holdout prevents participant leakage but is not an unbiased population sample, and P1–P3 are too few for defensible population-level significance tests or confidence intervals. Larger and more diverse samples with prespecified repeated grouped or leave-one-participant-out validation are needed. The archived records available for this revision also do not preserve a verifiable class-by-class count table for segmented holdout windows.

Third, several robustness questions were not quantified. The study did not define a quantitative similarity threshold, compute pairwise kinematic distances, or test confusion rate against similarity. Zero-padding was used for short executions, but padding sensitivity was not evaluated. The comparison was limited to SVC, RF, NB, and one LSTM configuration, so broader temporal, graph-based, or sequence-alignment benchmarks are needed to determine whether the observed model ranking generalizes.

Finally, sensing and deployment measures remain incomplete. Only the eight analyzed upper-limb IMUs were used for classification, without sensor ablation or systematic placement-perturbation testing. Inference latency and end-to-end real-time performance were not measured. Future work should therefore evaluate sensor reduction, placement robustness, multimodal fusion, class-specific performance, repeated participant-level validation, inference latency, and downstream SOP/HRC outcomes in realistic work settings.

## 6. Conclusions

This controlled pilot study provides two complementary empirical findings. First, a model developed from 13 participants retained strong aggregate recognition performance when evaluated on three entirely unseen challenge-holdout participants whose observed execution patterns and fluency differed comparatively from those of the model-development participants. Under the best RF configuration, aggregate accuracy reached 0.950 with an F1 score of 0.939, providing controlled evidence of participant-independent transfer across inter-individual execution variability. Second, this strong aggregate performance did not imply uniform class-level reliability, as prominent errors remained concentrated in S18–S19, S4–S11, and S12–S14. Thus, participant-independent transfer capability and local class separability represent complementary aspects of recognition performance.

The scientific contribution is therefore not a new algorithm or a claim that fine-grained industrial HAR has been solved, but empirical evidence about both the transfer capability and the remaining class-specific limitations of motion-only wearable-IMU recognition. The ability to retain substantial discrimination when applied to unseen individuals with comparatively different execution characteristics is an important methodological prerequisite for future AI-assisted work study and HRC. At the same time, the localized errors show why aggregate accuracy alone remains insufficient for evaluating finer-grained operational recognition. This study does not demonstrate detection of incorrect movements, SOP conformance, end-to-end industrial-process monitoring, or real-time HRC.

## Figures and Tables

**Table 1:** Synthesis of related HAR studies and their relevance to this research.

Study	Application	Recognition Focus/Representation	Recognition Scope	Relevance to This Study
Cheng et al. [1]	Logistics; wearable IMUs	Sequential operational elements	Small task-specific sets	Direct predecessor; participant-independent IMU recognition.
Cheng et al. [2]	Manual assembly; Kinect	DTW + KNN sub-actions	10 classes	Sequence alignment for SOP-defined sub-actions; manual segmentation.
Duarte and Neto [4]	Collaborative assembly	Event-camera primitives	5 classes	Unseen-subject HRC perception with a small vocabulary.
Benmessabih et al. [5]	Industrial assembly	3D-skeleton segmentation	12–14 labels	Similar gestures, variable duration, and boundary ambiguity.
He et al. [6]	Aviation assembly	Skeleton + RGB context	7 classes	Multimodal context improves similar-action discrimination.
Huang et al. [7]	Industrial procedures	Temporal segmentation	Procedure-specific	Adds boundary, redundant-behavior, and process-state uncertainty.
Patruno et al. [8]	Assembly	Projected skeletal distances	12 actions	Directional representation for subtle assembly motions.
Rajabi et al. [9]	Work assessment	RGB + temporal modeling	8 task classes	Temporal recognition/segmentation for occupational assessment.
Ding et al. [10]	Generic skeleton HAR	Graph convolution	60/120 classes	Large multiclass scale; not an industrially adjacent label set.
Ryu et al. [11]	Fine-grained sports	Sequential object context	Fine-grained activities	Shows the potential value of contextual object information.

**Table 2:** Characteristics of the participant-level model-development dataset before fixed-window segmentation.

Model-Development Participants	Movement Classes	Samples	Mean Length (Frames)	SD (Frames)	Minimum (Frames)	Maximum (Frames)
13	22	279 *	185	48.2	36	330

* The asterisk indicates that some originally collected records were unavailable because of sensor interruptions; affected records were removed before segmentation and model development.

**Table 3:** Candidate segmentation windows evaluated for the variable-duration fundamental-movement recordings.

Window Setting	Length (Frames)	Approximate Basis
Window 1	62	One-third of mean sample length
Window 2	93	One-half of mean sample length
Window 3	124	Two-thirds of mean sample length

**Table 4:** Hand-crafted window-level features extracted for the conventional classifiers.

Feature	Definition
mean	Arithmetic mean of each variable within the segmented window
median	Median of each variable within the segmented window
abs_energy	Sum of squared values for each variable
std	Standard deviation of each variable within the segmented window
var	Variance of each variable within the segmented window
min	Minimum value of each variable within the segmented window
max	Maximum value of each variable within the segmented window
skew	Skewness of each variable within the segmented window
kurt	Kurtosis of each variable within the segmented window
mse	Mean spectral energy for each variable within the segmented window
mnx	Number of mean crossings for each variable within the segmented window

**Table 5:** Classifier implementations and principal training settings used in the fundamental movements S1–S22 comparison.

Model	Principal Settings
SVC	scikit-learn SVC; linear kernel selected after comparing linear, RBF, and sigmoid kernels
RF	scikit-learn RandomForestClassifier; entropy criterion; n_estimators and max_depth selected using GridSearchCV
NB	scikit-learn GaussianNB; default Gaussian naive Bayes implementation used in the reported analysis
LSTM	TensorFlow/Keras; 64 LSTM units; dropout 0.1; three Dense layers (256 units each); Adam, learning rate 0.001; batch size 100; up to 300 epochs; cross-entropy loss

**Table 6:** Best holdout configuration for each classifier across scaling strategies and candidate window lengths.

Classifier	Best Scaling	Best Window (Frames)	Accuracy	F1	Training Time (s)
SVC	Min–max	124	0.778	0.710	3.9
RF	Maximum-absolute	124	0.950	0.939	10.9
NB	Maximum-absolute	93	0.663	0.602	3.1
LSTM	Maximum-absolute	124	0.644	0.591	676.3

**Table 7:** Participant-level classification performance using min–max scaling. The reported window is the best window for each classifier under this scaling method.

Model	SVC		RF		NB		LSTM	
Best window length (frames)	124		124		93		93	
Index	Accuracy	F1	Accuracy	F1	Accuracy	F1	Accuracy	F1
P1	0.745	0.640	0.787	0.685	0.393	0.336	0.506	0.407
P2	0.667	0.573	0.788	0.748	0.478	0.449	0.449	0.407
P3	0.923	0.917	0.923	0.912	0.670	0.641	0.857	0.848
Avg.	0.778	0.710	0.833	0.782	0.514	0.475	0.604	0.554
Std.	0.107	0.149	0.064	0.096	0.116	0.126	0.180	0.208
Model training time (s)	3.9		12.9		3.0		641.9	

**Table 8:** Participant-level classification performance using maximum-absolute scaling. The reported window is the best window for each classifier under this scaling method.

Model	SVC		RF		NB		LSTM	
Best window length (frames)	124		124		93		124	
Index	Accuracy	F1	Accuracy	F1	Accuracy	F1	Accuracy	F1
P1	0.745	0.652	0.979	0.939	0.438	0.374	0.511	0.479
P2	0.667	0.580	0.909	0.927	0.638	0.530	0.576	0.490
P3	0.923	0.896	0.962	0.952	0.912	0.901	0.846	0.806
Avg.	0.778	0.709	0.950	0.939	0.663	0.602	0.644	0.591
Std.	0.107	0.135	0.030	0.010	0.194	0.221	0.145	0.151
Model training time (s)	3.7		10.9		3.1		676.3	

## Data Availability

The dataset is available from the authors upon request.
